# The Effects of Noise and Heat Strain on the Work Ability Index (WAI) among Rubber Factory Workers

**DOI:** 10.5334/aogh.2515

**Published:** 2019-07-03

**Authors:** Reza Kazemi, Zahra Zamanian, Maryam Khalifeh, Rasoul Hemmatjo

**Affiliations:** 1 Department of Ergonomics, School of Health, Shiraz University Of Medical Sciences, Shiraz, IR; 2 Department of Occupational Health, School of Public Health, Urmia University of Medical Sciences, Urmia, IR

## Abstract

**Introduction:**

Assessing the work ability and factors affecting it is essential in developing strategies for preventing damages and managing risks. This study aimed to investigate the simultaneous effect of noise level and physiological strain as well as individual characteristics on the work ability.

**Method:**

The population in this cross-sectional study included workers of a rubber factory. The TES noise dosimeter was used to examine individual exposure and the electro polar RS100 was used to measure physiological strain index (PSI). Individual characteristics and the work ability index were evaluated using the WAI questionnaire and analyzed using SPSS version 19. chi-square test, pearson correlation coefficient, and one-way and multiple ANOVA were used for data analysis.

**Results:**

The final modeling showed that age, exercising period, Equivalent Continuous Sound Pressure Level (Leq), PSI and employment status had significant correlations with the work ability index (p < 0.05). The modified r^2^ for the obtained model was also calculated to be 0.483.

**Discussion and conclusion:**

Based on the findings, number of exercising hours, employment status, age, Leq, and psi are among the factors affecting the work ability index. Use of management and engineering controls are recommended to balance work environments exposed to noise and heat and improve the work ability index. Further, improving employment status due to creating a sense of stability and reducing stress as well as enhancing lifestyle quality can be effective in increasing the work ability index.

## Introduction

Work ability is defined as the degree to which workers are able to adapt to their occupational needs physically or mentally based on their health level [1,2,3].

From the perspective of occupational health, the concept of work ability is based on the balance between individual characteristics and occupational needs [4]. Work ability is a complex and dynamic process, reflecting the interactions between the physical and mental ability (individual characteristics), working conditions, employees’ functional capabilities, employees’ health status, as well as the individual’s assessment of their position in the organization and society (social characteristics) [5]. According to the research conducted by Ilmarinen and Tommy (2004), the work ability is defined as follows: the worker’s ability to perform their work based on their mental and physical conditions and occupational needs and also the capacities and capabilities of individuals in relation to the physical and psychological needs of work [6]. Assessing work ability is important in defining ways to prevent damages and manage risks. Therefore, many studies have been carried out on assessment of work ability and factors affecting it. Such studies have shown that individual, psychosocial, lifestyle and occupational factors influence work ability [7]. Some studies have investigated occupational factors associated with work ability [8,9]. The results of these studies have shown that there is a significant correlation between work-related risk factors and work ability. Heat is one of the work-related risk factors, which can lead to a heat strain (body heat response) such as increased heart rate and body temperature [10]. Exposure to heat strain, in addition to creating health risks, can lead to loss of work efficiency [8,11]. The results of previous studies indicate that the work ability index is reduced due to exposure to heat at the work environment. Noise is another prevalent work-related risk factor at the work environment. Over 600 million people in the world are exposed to noise level above the standard (85 dB) at their work environment [12,13], which is considered as a major risk in many work environments [14]. Although there is no direct study to assess noise effect on work ability, studies have shown that reducing productivity and increasing human error and fatigue are among the noise exposure consequences at the work environment, in addition to the physiological effects of noise on humans [3].

In general, the results of previous studies indicate that the work ability index is affected by individual factors and the context of the individual’s presence. Therefore, to determine factors influencing work ability, it is necessary to study work-related factors along with individual factors.

In previous studies, the effect of noise at the work environment has not been directly investigated on the work ability index. Similarly, the simultaneous effect of exposure to noise and heat has not been studied on work ability [15]. Due to the prevalence of these two factors in many occupations, evaluating their simultaneous effect and determining the separate load of each factor, along with individual characteristics and lifestyle, can help to formulate more comprehensive prevention programs. Therefore, the aim of this study was to determine the impact of noise and heat as two prevalent work-related risk factors as well as individual characteristics and lifestyle on the work ability index.

## Method and instruments

This cross-sectional study was carried out in an Iranian rubber factory with a population of 670. The study population was 197 production line workers in the factory, who were randomly selected based on the inclusion criteria by considering α = 0.05, power of 90%, and r = 0.27. The inclusion criteria were work experience over two years, acceptable physical and mental health, non-smoking or non-addiction, and consent to participate in the study. This study was conducted in accordance with the ethical guidelines established by Shiraz University of Medical Sciences. In order to observe ethical issues, informed consent was obtained from all the participants prior to the experiment.

## The demographic questionnaire

According to the factors considered for the study, a questionnaire was used, containing questions about demographic features (height, weight, age, work experience, marital status, etc.). Exercising as an index in lifestyle was also determined by the same questionnaire.

## Measurement of the work ability index

The reliable Persian version of the WAI index was used to assess the work ability index. The psychometric properties of P-WAI were reported by Abdolalizadeh et al. [7] This index is developed by researchers from the Finnish Institute of Occupational Health and measure seven dimensions including: 1. current work ability in comparison with the best living time, 2. work ability in relation to occupation nature, 3. current illnesses detected by the physician, 4. individual estimation of work-related impairments, 5. sick leave during the past 12 months, 6. work ability prediction in the next two years, and 7. mental capabilities.

Based on the rating of these dimensions by the subject being studied, the final score assigned to each worker will be between 7–49, with 49 being the best estimate of work ability and 7 being the worst estimate of work ability. Accordingly, the ability of people can be classified into four categories:

1. The score 1–27: weak work ability
2. The score 28–36: moderate work ability
3. The score 37–43: good work ability
4. The score 44–49: outstanding work ability

## Measurement of the physiological strain index (PSI)

To measure this index, it is necessary to measure heart rate during both rest and activity as well as inner ear temperature during both rest and activity. Electro-Polar RS100 was used to measure heart rate and ear canal temperature. For this purpose, the device sensor was connected to the worker’s chest and the device receiver, which is similar to a wristwatch, was closed around the worker’s wrist. After installing the devices and after 30 minutes of rest in a cool room (WBGT = 23.6 + 1.4) [16], the mean heart rate and ear canal temperature were recorded at rest by the POLYGREEN device placed in the ear canal. Then, heart rate and ear canal temperature were recorded at 20, 40, and 60 minutes after executing tasks and calculated according to the following formula. Finally, the mean score of the beginning and end of the shift was recorded as the final score [17].

{\rm{PSI }} = 5({{\rm{T}}_{{\rm{rest}}}} + {{\rm{T}}_{{\rm{rec}}}})(39.5 - {{\rm{T}}_{{\rm{rec}}}}) - 1 + 5({\rm{H}}{{\rm{R}}_{\rm{t}}} - {\rm{H}}{{\rm{R}}_{\rm{0}}})(180 - {\rm{H}}{{\rm{R}}_{\rm{0}}}) - 1

HR_0_: Heart rate at rest (before beginning activity)

HR_t_: Heart rate during activity

T_rec_: Deep temperature during activity

T_res_: Deep temperature at rest

## Measurement of noise exposure level

The TES Noise Dosimeter was used to measure individual exposure. The dosimeter is the most reliable method for evaluating individual exposure. However, because of the high cost and time consuming nature of this method and given that the individual exposure pattern of the workers had a certain periodic frequency, a short 20-minute time period was used and extended to the total exposure time [18]. According to ISO 9612, the microphone was placed at a distance of 4 cm from the top of the shoulder in order to prevent individual effects [19]. We used the following formula to estimate the 8-hour equilibrium with respect to a short-term dosimeter.

\frac{{T2}}{{T1}} = \frac{{D2}}{{D1}}

T2: Individual work shift (six hours)

T1: Measurement duration (20 minutes)

D2: The calculated dose for a six-hour work shift (%)

D1: The measured dose in 20 minutes (%)

Then, we converted the dose amount measured by the dosimeter (in percent), using the diagram accompanying this device, to an equivalent level (eight-hour Leq in dB) [18].

## Data analysis methods

The SPSS software version 19 was used to analyze the collected data. In order to describe the quantitative variables, the “mean” and “standard deviation” were used to show the central tendency and index of dispersion, respectively. Moreover, the Chi-square test was used to determine the degree of independence or dependence between two qualitative variables, and also to examine and compare the frequency distribution of qualitative variables such as education level and shift work system in people with high and low work ability levels. Additionally, the Pearson correlation coefficient test was used to determine the linear correlation between two quantitative variables. The correlation between a dependent variable and an independent variable was analyzed using one-way ANOVA. Finally, a multiple linear regression analysis was used to determine the final model.

## Results

Table 1 shows the demographic features of the studied population. The mean age and work experience as two important parameters in this study were 32.82 and 10.7 years, respectively.

**Table 1:** Individual characteristics of the studied population and their environmental exposure values.

Demographic features	Index			

	Mean	SD	Min	Max

**Height (cm)**	175.95	6.32	156	195
**Weight (kg)**	77	8.89	58	110
**BMI (Kg/m^2^)**	24.89	2.69	18.5	34.6
**Age (year)**	32.82	5.61	20	46
**Work experience (year)**	10.17	5.13	1	20
**Exercise duration per week (hours)**	2.72	2.81	0	10
**Marital status**	Single	*37	**8.18	
	Married	*160	**2.81	
**Education**	Diploma and lower	*165	**83.7	
	Associate degree	*27	**13.7	
	Bachelor’s degree and higher	*5	**2.5	
**(dB)Leg**	87.32	2.35	83.45	94.31
**PSI**	2.55	0.65	1.28	4.25
**WBGT**	27.91	4.07	21.5	37.5

* Number. ** Percentage.

Tables 2 and 3 shows the correlation coefficient between the demographic and environmental variables with the WAI index in the studied subjects. There is a statistically significant relationship between age, employment status, education exercise Dosimetric index and PSI with WAI (p < 0.05). However, the correlation between the mean work ability score and body mass index (BMI) was not significant (p < 0.05). Although this correlation was not significant, it was negative and the correlation coefficient was –0.28. This means that there was an inverse linear correlation between these two variables and thus increasing BMI was accompanied by reduced work ability.

**Table 2:** The correlation between the demographic variables and work ability index.

Variable		Work ability								*P-Value

		Weak		Moderate		Good		Outstanding		

		Frequency	Percent	Frequency	Percent	Frequency	Percent	Frequency	Percent	

Marital status	Married	30	18.8	86	53.8	37	23.1	7	4.4	0.163
	Single	5	13.5	17	45.9	10	27	5	13.5	
Age	28–20	1	1.8	18	32.1	26	465.4	11	16.9	0.0001
	37–29	23	22.8	60	59.4	17	16.8	1	1	
	46–38	11	28.2	24	61.5	4	10.3	0	0	
Employment status	Permanent contract	23	88.5	–	–	–	–	3	11.5	0.05
	Fixed-term contract	115	67.3	–	–	–	–	56	32.7	
Education	Diploma and lower	29	17.6	85	51.5	40	24.2	11	6.7	0.078
	Associate degree and higher	6	18.8	18	56.4	7	21.9	1	3.1	
Exercising	Yes	9	8	57	50.4	37	32.7	10	8.8	0.0001
	No	26	31	46	54.8	10	11.9	2	2.4	

* The Chi-square test.

However, the Leq and WAI indices showed a strong significant negative correlation at the value of 0.698. This means that increasing Leq was correlated with a decrease in WAI.

Moreover, the correlation was statistically significant between work ability and PSI. The correlation was observed to be negative at the value of 0.285, showing a weak correlation between these two variables. This means that higher heat strain caused lower work ability index.

Based on the results of the Chi-square and one-way ANOVA tests (Tables 2 and 3), the variables of work experience, exercising, age, employment status, marital status, education, BMI, Leq and PSI were eligible to enter the model.

**Table 3:** The correlation between the studied variables and work ability index.

Variables	WAI	

	The correlation coefficient	P-Value

BMI	–0.128	0.078
Dosimetric index	–0.402	0.001
PSI	–0258	0.001

**Table 4:** Factors affecting work ability using the linear regression model in the studied subjects (n = 197).

Variables	B	Std- Error	Beta	T	Sig

Age	–0.629	–0.074	–0.545	–8.514	0.000
Exercising time	0.549	0.125	0.244	4.383	0.000
Leq	–0.630	0.151	–0.229	–4.165	0.000
PSI	–2.141	0.544	–0.218	–3.939	0.000
Employment status	–2.527	1.267	–0.132	–0.934	0.048

The results of regression analysis of factors affecting work ability in the studied subjects are presented in Table 4. Modeling results showed that age, exercising time, Leq, PSI and employment status had a significant correlation with work ability (p < 0.05). The modified R^2^ for the obtained model was equal to 0.483, indicating that 48.3% of the variation in the response variable was predicted by the model. According to the t statistic, the most important variables were age, exercising hours (weekly) and Leq, respectively.

{\rm{WA }} = 117/749 - (0/629{\rm{ age) }} + (0/549{\rm{ exercising time}}) - (0/63{\rm{ Leq}}) - (2/141{\rm{ PSI}}) - (2/527{\rm{ employment status}})

## Discussion

The purpose of this study was to investigate the effect of exposure to noise and heat at the work environment along with individual characteristics on work ability in a rubber factory. Although previous studies have examined the effects of each of the above variables separately, there was no study addressing the simultaneous effects of exposure to noise and heat as two prevalent work-related risk factors on work ability [10]. Therefore, this study was the first of its kind to examine the simultaneous effects of individual characteristics as well as exposure to noise and heat on work ability, and to model the individual and occupational variables influencing the work ability index [20].

The results of this study indicated the moderate work ability index in the studied population, which is similar to the studies conducted in other industries in Iran [20]. On the other hand, the work ability index was moderate to weak in more than half of the studied population, which is concerning given the relatively young nature of the studied population. The reason is that, according to the definitions, the work ability index demonstrates the adaptation between needs and work ability, and these results indicated the unfavorable proportions between occupational needs and abilities and capacities of the individuals under study [20].

Individual characteristics (physical and psychological) are important parameters affecting the work ability index [20,21]. In the present study, the results also showed that some individual variables including age and work experience had an impact on individuals’ work ability. Several studies have confirmed the correlation between age and work ability [21]. In general, it can be claimed that a majority of studies are in agreement with regard to the effect of age on the work ability index, implying that the work ability index decreases with aging. This can be imposed by reduced physical and cognitive capacities [22].

In this study, education level had no significant correlation with the work ability index. In some studies, the correlation between education level and work ability is documented as such the higher levels of education lead to improved work ability [5,21]. In some other studies, there is no correlation between work ability and education level [23,24]. On the other hand, although the effect of BMI on the work ability index was not significant, these two variables were inversely correlated, indicating that the work ability index decreases with increasing BMI. These results are consistent with the findings of other studies.

Due to the diversity of lifestyle assessment indices, exercising was examined as one of the most important lifestyle indices in this study. The mean exercising period (weekly) showed statistically significant differences in the four groups of work abilities. This means that increasing the exercising period (weekly) among individuals improved their work ability, and higher work ability was recorded for those who spent more hours on exercising. These findings are consistent with Gouldilp’s findings. In order to justify this finding, it can be argued that exercising can affect work ability in two ways. First, it is well-connected to health so that those who spend more time on exercising are healthier. Second, exercising improves individuals’ capabilities and potentials, which is correlated with their work ability. Narszalek (2005) found that working in a warm environment could increase the physiological rate (deep temperature and heart rate) in middle-aged individuals and reduce their work ability [25]. In his study, Tommy (2001) reported an inverse correlation between work ability and heat strain [26].

There was a significant negative and moderate correlation between work ability and dosimetry indices. This means that higher noise exposure led to lower work ability index. This finding can be justified by the findings obtained in other studies. Vandeberg (2009) found that high physical workloads and inappropriate physical environments were important factors reducing work ability [27]. Noise causes distress in individuals [28] and has consequences such as heart disease, mental impairment, and anxiety disorders, leading to disturbed work ability and early retirement [28]. Hence, noise reduces individuals’ physical abilities and capacities and also threatens their health status, as two factors decreasing the work ability index [29].

The final model revealed that age, exercising, employment status, and exposure to noise and heat were factors affecting work ability. In this model, aging and increased exposure to noise and heat in the work environment had a negative effect on the work ability index; however, increasing exercising period improved individuals’ work ability. Moreover, individuals with permanent employment contract in the organization had a better work ability index. The model can well predict the work ability index in environments exposed to noise and heat. The model can also indicate that exercising is considered as an acceptable variable for the work ability index such that permanence in employment status leads to more desirable work ability index.

## Conclusion

Physical conditions are of greater importance in the indoor environment of factories and workshops than in the outdoor environment from the viewpoint of health, convenience, and impact on individuals’ performance and work ability since individuals spend great time in these places. Work ability describes individuals’ capacities and capabilities with regard to physical and psychosocial needs, and is a useful tool for identifying those at risk of imbalance between health, ability, and occupational needs. Studies have also revealed that exposure to risk factors has a negative impact on individuals’ health, safety, productivity, and work ability.

According to the findings, number of exercising hours, employment status, age, Leq, and PSI are among the factors affecting the work ability index. Use of management and engineering controls are recommended to balance work environments exposed to noise and heat and improve the work ability index. Further, improving employment status due to creating a sense of stability and reducing stress as well as enhancing lifestyle quality can be effective in increasing the work ability index.
